# Ectopic adrenocortical adenoma in the abdominal wall linked to the head of the pancreas: A case report

**DOI:** 10.1016/j.ijscr.2024.110568

**Published:** 2024-11-08

**Authors:** Ghena Alhadwah, Nahar Ismaiel, Jaafar Shater, Ali Daoud, Maen Haidar

**Affiliations:** a Department of Surgery, Tishreen University Hospital, Latakia, Syria; b Department of Pathology, Tishreen University Hospital, Latakia, Syria; c Faculty of Medicine, Tishreen University, Latakia, Syria

**Keywords:** Ectopic adrenocortical adenoma, Abdominal wall, Surgery, Case report

## Abstract

**Introduction:**

Ectopic adrenocortical adenomas are infrequent adrenal tumors that deviate from their usual location. Herein, we report a case of an ectopic black adrenocortical adenoma situated in the abdominal wall, intricately linked to the head of the pancreas.

**Case presentation:**

A 27-year-old female presented to the emergency room with severe right iliac pain, nausea, vomiting, and high fever. Clinical exam showed signs of acute appendicitis in addition to distinctly outlined, slightly painful, and immobile mass located in the right hypochondrium. Both the inflamed appendix and mass were removed surgically. Pathological examination of said mass with subsequent immunohistochemical staining confirmed the diagnosis of an ectopic adrenocortical adenoma.

**Discussion:**

Our case is unique as it occurred in an adult female which is rare for these types of lesions. In addition to that the lesion was located in the anterior abdominal wall intimately linked with the head of the pancreas making this case the first documented case of such a location in the medical literature.

**Conclusion:**

The successful diagnosis and management of this rare presentation underscore the significance of a collaborative and multidisciplinary approach.

## Highlights

• Ectopic adrenocortical adenomas originate from small adrenal cortex fragments during development
• These lesions are rare in adults, especially women
• this is the first case of its kind reported in the literature

## 1 Introduction

Ectopic adrenocortical adenomas originate from small adrenal cortex fragments during development [1]. They can manifest in mesoderm-derived organs, such as the kidney and liver [5]. Most ectopic adrenocortical adenomas are non-functional. However, a small portion of these tumors are functional thus manifesting as different clinical syndromes depending on the type of secreted hormone [11]. Reported incidence rates in different areas include the celiac axis (32 %), broad ligament (23 %), kidney (0.1 %–6 %), and testicular adnexa (7.5 %) [6]. This distribution makes differentiation from tumors challenging without histological examination [5]. The phenomenon is often found on the right side and rarely on both sides [7]. Moreover, it is rare in adults, especially women [5]. Despite these known patterns, ectopic adrenal cortical adenomas rarely involve the abdominal wall. In this report, we present a case of a patient with an asymptomatic ectopic black adrenocortical adenoma in the anterior abdominal wall linked to the head of the pancreas. This case report has been reported in line with the SCARE criteria [14].

## 2 Case presentation

A 27-year-old female patient presented to the emergency room with severe right iliac pain, accompanied by nausea, vomiting, and high fever (40 °C), her other vital signs were within the normal range. The patient had regular menses and no known past medical history, obstetric and gynecological history or medication history. The physical examination showed a tender and immobile mass located in the right hypochondrium. Positive findings for McBurney's sign, Rovsing's sign, and Murphy's sign were noted. The patient appeared otherwise normal. Finally, we note that the patient did not suffer from symptoms associated with steroid excess (acne, striations, central obesity, irregular menstruation). Laboratory studies indicated a hemoglobin level of 9.4 g/dl, neutrophils at 73.1 %, prothrombin time (PT) of 98 s, albumin of 2.6 g/dl, and a C-reactive protein (CRP) level of 58 mg/dl. Other laboratory tests were within the normal range including adrenocortical hormones and adrenocorticotropic hormone (ACTH). An abdominal ultrasound scan was ordered to examine the gallbladder which appeared normal with no gallstones or signs of inflammation. However, the scan was unable to determine the nature of the abdominal mass nor was it able to rule out acute appendicitis. Thus acute appendicitis was suspected.

**Fig. 1:**
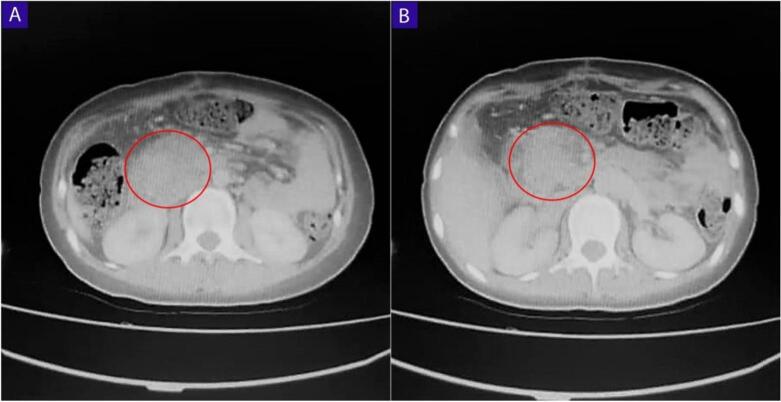
Abdominal CT scan (A and B) axial sections, a well-defined cyst (red circle), measuring 10 cm in the right hypochondriac fossa adhering to the head of the pancreas. (For interpretation of the references to color in this figure legend, the reader is referred to the web version of this article.)

**Fig. 2:**
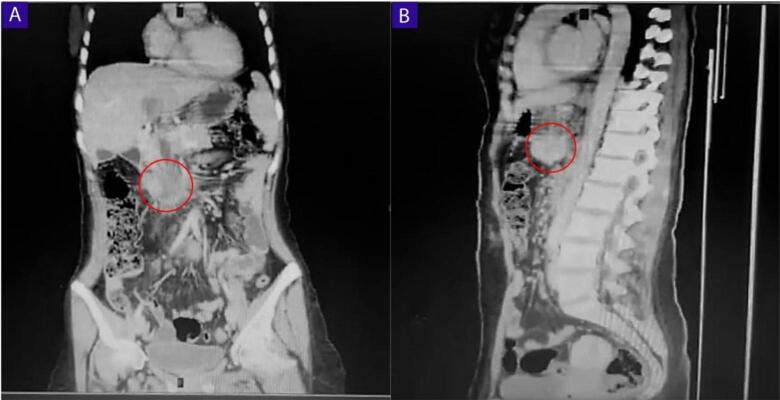
A Abdominal CT scan, coronal section, the cyst can be seen adjacent to the lower vena cava. B Abdominal CT scan, sagittal section, The cyst can be seen adjacent to the lower vena cava.

A CT scan was ordered and it showed a hyper-dense well defined cyst measuring 12 × 10 cm in the right hypochondriac region. The cyst appeared adhered to the head of the pancreas adjacent to the lower vena cava [Figs. 1, 2].

The medical team opted for an exploratory laparotomy to address both the suspected acute appendicitis and the abdominal cyst. Pre-operatively a blood transfusion was performed to elevate the patient's hemoglobin level (which reached 12 g/dl). Under general anesthesia a midline incision was made and sufficient dissection was performed until an enlarged and inflamed appendix with a thickened wall was discovered. The appendicular mesentery was ligated and the appendix was resected.

**Fig. 3:**
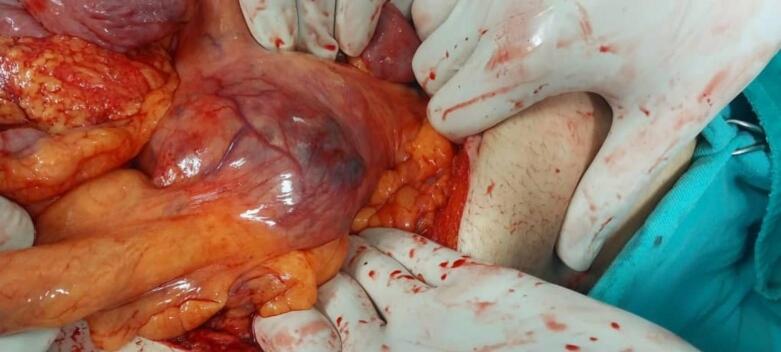
Surgical image, the cyst is visible.

[Fig. 3]. The abdomen was further explored and abdominal structures were manipulated until the cyst was located. Surgical dissection led to the unintended rupture of the cyst, releasing necrotic contents accompanied by heavy bleeding. Hemostasis was subsequently achieved, blood was transfused the surgical field was sufficiently cleaned, and a sample was obtained for pathological study.

The cyst was fenestrated and resected successfully in a piecemeal fashion. After the operation the patient felt well and all her symptoms subsided. Upon follow-up she remains disease free.

**Fig. 4:**
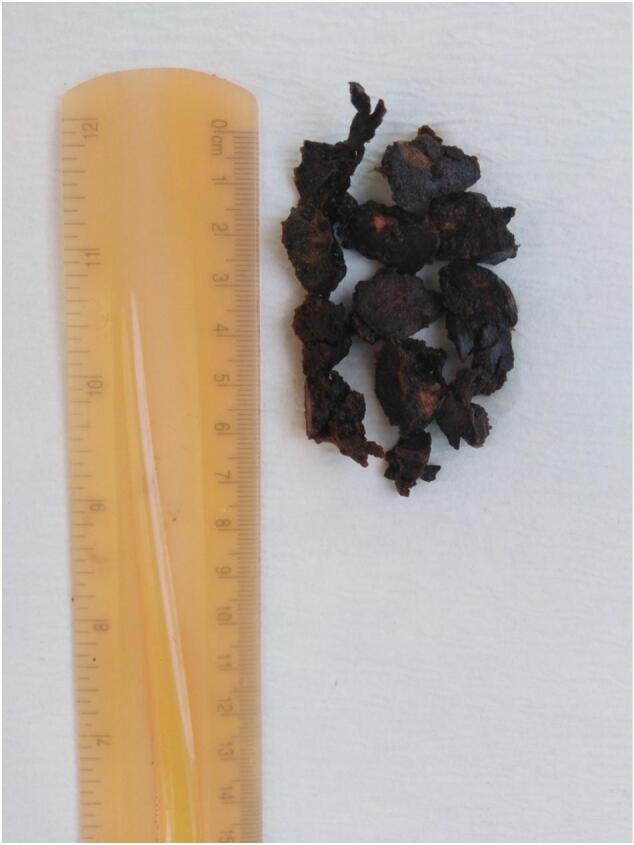
Gross specimen, multiple friable fragments, ranging in color from black to brown, measuring 7 cm in aggregate. (For interpretation of the references to color in this figure legend, the reader is referred to the web version of this article.)

**Fig. 5:**
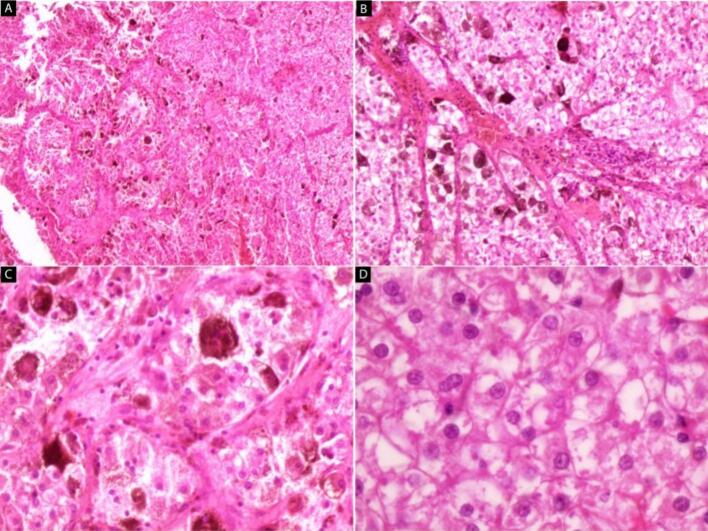
A Ectopic adrenocortical adenoma, cords, and clusters of cells with brown pigmentations, (H&E 40×). B Ectopic adrenocortical adenoma, cords, and clusters of cells with brown pigmentations, (H&E 100×). C Ectopic adrenocortical adenoma, Heavy deposits of golden-brown, slightly refractile granular pigments were observed (H&E 200×). D Ectopic adrenocortical adenoma, large, polygonal cells with clear to eosinophilic cytoplasm and centrally rounded nuclei (H&E 400×). (For interpretation of the references to color in this figure legend, the reader is referred to the web version of this article.)

Gross examination revealed multiple friable fragments, ranging in color from black to brown, measuring in aggregate 7 cm [Fig. 4]. Microscopic examination revealed large polygonal cells with clear to eosinophilic cytoplasm and centrally rounded nuclei. Additionally, heavy deposits of golden-brown, slightly retractile granular pigments were observed [Fig. 5]. The histopathological findings were consistent with a tumor of adrenocortical origin, but metastases or melanoma or pancreatic tumors should be ruled out; therefore, immunohistochemical staining (CK cocktail, CK20, and melanoma cocktail) were performed, and they were negative. A postoperative CT scan was conducted to determine the lesion's origin, revealing no other masses with similar characteristics. The final diagnosis of an ectopic adrenocortical adenoma was made.

## 3 Discussion

Ectopic adrenocortical adenomas are a rare phenomenon that usually happens when fragments of adrenal tissue shed off during adrenal development [3]. Some researchers like Anderson et al. postulated that adrenal ectopy may arise from pluripotent stem cells [13]. It is likely that an involution occurs within the first year of life which explains the prevalence of adrenal ectopy in young children [3,5].

Most ectopic adrenocortical adenomas are non-functional as they do not secrete any hormones. Due to that these tumors remain clinically silent and are usually incidentally detected. However some of these tumors secrete different hormones such as cortisol, aldosterone or androgens which in turn may cause patients to present with symptoms of Cushing's syndrome, hyperaldosteronism or virilization [11]. In our case the patient did not suffer from symptoms suggesting an increase in steroid secretion (including cortisol, aldosterone and androgens) and laboratory test showed normal levels of adrenocorticotropic hormone (ACTH) and adrenocortical hormones which confirmed the non-functional nature of the tumor.

These lesions are typically, asymptomatic and are usually discovered during childhood groin surgeries or other surgeries in adults, especially in regions linked to gonadal development, such as the spermatic cord [[2], [3], [4]]. Finally, they are most commonly located in the celiac axis (32 %), broad ligament (23 %), kidney (0.1 %–6 %), and testicular adnexa (7.5 %) and are usually asymptomatic [6].

Our case was unique as it occurred in a 27-year-old woman which is an exceedingly rare presentation. In addition to that the location of the ectopic adenoma is another rare occurrence as ectopic adenomas rarely involve the abdominal wall. Finally, the identification of an ectopic adrenocortical adenoma in the anterior abdominal wall particularly one linked to the head of the pancreas represents a rare and intriguing clinical scenario. We searched the related literature extensively and, to our knowledge, this is the first case of its kind reported in the literature underscoring the importance of reporting the unique anatomical location of the tumor to contribute to the expanding body of medical knowledge.

Preoperative diagnosis of these lesions is challenging due to the absence of specific clinical and radiological features [11]. However, radiological imaging, including contrast-enhanced CT and magnetic resonance imaging (MRI), can provide valuable information in characterizing the lesion and its relationship with adjacent structures [11].

The most common differential diagnoses for ectopic adrenocortical adenomas include hemangioma, melanoma, and metastatic lesions [10]. While pancreatic tumors may not exhibit this particular appearance, they should be considered as a differential diagnosis, as our case involves a tumor located adhering to the head of the pancreas.

Pathological examination is the golden standard for diagnosing these lesions and differentiating them from other tumors [5]. It usually demonstrates characteristic features of adrenal cortical adenomas, such as clear cell cytoplasm, absence of mitotic figures, and low proliferative activity [12].

Finally, surgical resection remains the preferred modality of treatment for adrenocortical adenomas even when located in extra-adrenal sites [12].

## 4 Conclusion

Ectopic adrenocortical adenomas are infrequent tumors that arise from adrenocortical fragments. Herein we present a unique instance of an ectopic adrenocortical adenoma located in the abdominal wall and intricately linked to the head of the pancreas. The successful diagnosis and management of this rare presentation underscore the significance of a collaborative and multidisciplinary approach. As the first documented case of its kind, this report contributes to the expanding knowledge base of ectopic adrenal tumors and serves as a reminder for clinicians to consider rare entities in the differential diagnosis, facilitating timely and effective management. Continued research and reporting of such cases is crucial for advancing our understanding of these rare tumors and optimizing patient care.

## Author contribution

All authors contributed to this manuscript.

Ghena Alhadwah: Writing - original draft, reviewing, and editing.

Nahar Ismaiel: Writing - original draft, reviewing, and editing.

Jaafar Shater: Writing - original draft, reviewing, and editing.

Ali Daoud: Reviewing and editing.

Maen Haidar: Supervision; final reviewing and editing.

## Consent

Written informed consent was obtained from the patient for publication of this case report and accompanying images. A copy of the written consent is available for review by the Editor-in-Chief of this journal on request.

## Ethical approval

Given the nature of the article, a case report, no ethical approval was required.

Name of institution: Tishreen University.

## Guarantor

Prof. Maen Haidar.

## Research registration number

N/A.

## Provenance and peer review

Not commissioned, externally peer-reviewed.

## Funding

No funding was needed.

## Conflict of interest statement

The authors declare no conflict of interest.

## References

[1] Schechter D.C. (Mar 1968). Aberrant adrenal tissue. Ann. Surg..

[2] Kim T.-H., Cha D.-S., Han K.-H., Youm H.-S., Hyon N.N., Chong Y.-S. (2008). Ectopic adrenal tissue in right uterine adnexa: a case report. Obstet. Gynecol. Sci..

[3] Senescende L., Bitolog P.L., Auberger E., Zarzavadjian Le Bian A., Cesaretti M. (2016). Adrenal ectopy of adult groin region: a systematic review of an unexpected anatomopathologic diagnosis. Hernia.

[4] Chew K.T., Abu M.A., Arifuddin Y., Mohamed Ismail N.A., Nasir N.A.M., Mohammed F., Nur Azurah A.G. (2017). Ectopic adrenal tissue associated with borderline mucinous cystadenoma of ovary: a case report with review of the literature. Horm. Mol. Biol. Clin. Invest..

[5] Sugiyama T., Tajiri T., Hiraiwa S., Inomoto C., Kajiwara H., Kojima S., Tobita K., Nakamura N. (2015). Hepatic adrenal rest tumor: diagnostic pitfall and proposed algorithms to prevent misdiagnosis as lipid-rich hepatocellular carcinoma. Pathol. Int..

[6] Kassaby S.S., Velilla R.E., Shurbaji M.S. (2017). Adrenal cortical heterotopia in an inguinal hernia sac of an adult: a case report and literature review. Hum. Pathol.: Case Rep..

[7] Mares A.J., Shkolnik A., Sacks M., Feuchtwanger M.M. (Jun 1980). Aberrant (ectopic) adrenocortical tissue along the spermatic cord. J. Pediatr. Surg..

[10] Fonseca J.J.S., Pirola S. (2012). Black adenoma of the adrenal gland. Int. J. Surg. Pathol..

[11] Endo Masashi, Fujii Hiroyuki, Fujita Akifumi, Takayama Tatsuya, Matsubara Daisuke, Kikuchi Tomohiro, Manaka Saki, Mori Harushi (2022). Ectopic adrenocortical adenoma in the renal hilum mimicking a renal cell carcinoma. Radiol. Case Rep..

[12] Lu D., Yu N., Ma X., Zhang J., Guo X. (2018). An ectopic adrenocortical adenoma in renal hilum presenting with Cushing’s syndrome: a case report and literature review. Medicine.

[13] Anderson J.R., Ross A.H.M.L. (1980). Ectopic adrenal tissue in adults. Postgrad. Med. J..

[14] Sohrabi C., Mathew G., Maria N., Kerwan A., Franchi T., Agha R.A. (2023). The SCARE 2023 guideline: updating consensus Surgical CAse REport (SCARE) guidelines. Int. J. Surg. Lond. Engl..

